# Pulmonary Hypertension Associated With Interstitial Lung Diseases

**DOI:** 10.1016/j.chest.2025.07.4107

**Published:** 2025-09-10

**Authors:** Arun Jose, Namita Sood, Jean M. Elwing, Bindu Akkanti, Abubakr Bajwa, Roberto Bernardo, Rodolfo A. Estrada, Munish Sharma, Francisco J. Soto, Adriano R. Tonelli, Divya Verma, Janine Vintch, Sandeep Sahay, Oksana A. Shlobin, Pulmonary Vascular Disease (PVD) Section

**Affiliations:** a University of Cincinnati, Cincinnati, OH; b University of California at Davis, Sacramento, CA; c McGovern Medical School, University of Texas Health at Houston, Houston, TX; d Ascension Medical Group, Jacksonville, FL; e Indiana University School of Medicine, Indianapolis, IN; f University of Texas Health at San Antonio, San Antonio, TX; g Baylor College of Medicine, Houston, TX; h University of Tennessee, Knoxville, TN; i Cleveland Clinic, Cleveland, OH; j Emory University School of Medicine, Atlanta, GA; k Harbor UCLA Medical Center, Los Angeles, CA; l Houston Methodist Hospital, Houston, TX; m Inova Heart and Vascular Institute, Fairfax, VA

**Keywords:** diagnosis, interstitial lung disease, pulmonary arterial hypertension, pulmonary hypertension, treatment

## Abstract

Interstitial lung disease (ILD) is a term encompassing a wide array of pulmonary conditions characterized by inflammation and fibrosis of the pulmonary parenchyma. Pulmonary hypertension (PH) is frequently encountered in patients with fibrotic ILDs and poses unique difficulties for both diagnosis and management. Patients with ILD-associated pulmonary hypertension (ILD-PH) are complex, often ailing and presenting with multiple comorbidities whose individual contributions to the underlying PH can be challenging to disentangle. Evidence supporting treatment with PH-specific medications in ILD-PH is limited. This edition of “How I Do It” presents a longitudinal case-based discussion of ILD-PH to address these challenges, highlight pearls and pitfalls in the diagnostic workup of these patients, and provide a framework for the practical evidence-based approach to accurate diagnosis and management of these challenging cases.

## Case, Part 1

A 60-year-old female with a medical history of hypertension and hyperlipidemia was evaluated by her primary care provider for concerns of progressive dyspnea on exertion. The patient reported a viral syndrome several weeks prior, with subacute worsening of dyspnea, and although her infectious symptoms resolved, the dyspnea persisted. She smoked 1 pack daily for 15 years, quitting a year ago. Her pulmonary function tests (PFTs) were notable for no obstruction, a moderate restrictive defect (FVC of 66% of predicted, total lung capacity of 67% of predicted), and a diffusion capacity for carbon monoxide (Dlco) of 38% of predicted. Her chest radiograph showed bilateral basilar reticular opacities.

**Figure 1:**
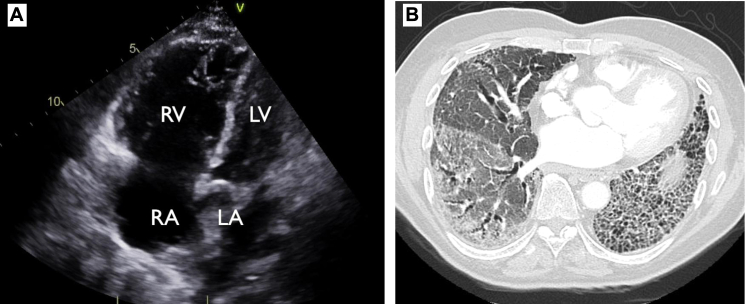
Initial diagnostic testing. Representative images of (A) transthoracic echocardiography apical 4-chamber view demonstrating enlargement of RA and RV, and (B) high-resolution chest CT demonstrating basilar-dependent ground-glass opacities and peripheral subpleural reticulations with honeycombing. LA = left atrium; LV = left ventricle; RA = right atrium; RV = right ventricle.

She was referred to a pulmonologist for further evaluation. On presentation she had a BP of 135/85 mm Hg, with oxygen saturation of 95% while breathing room air. Her physical examination was notable for scattered inspiratory “velcro” crackles audible along the lower lung fields and mild digital clubbing. Transthoracic echocardiography (TTE), plasma brain natriuretic peptide (BNP), noncontrast high-resolution chest CT scan, and 6-minute walk test (6MWT) were done for further evaluation. Her TTE demonstrated enlargement of the right ventricle (RV) and right atrium (RA) with normal left ventricular (LV) function (Fig 1A). The tricuspid valve was not well visualized, and right ventricular systolic pressure (RVSP) could not be estimated. Her BNP level was elevated at 286 pg/mL (reference range, < 100 pg/mL). CT scan of the chest showed an enlarged pulmonary artery at 4.2 cm and both ground-glass opacities and peripheral subpleural reticulations with honeycombing in a basilar distribution (Fig 1B). The 6MWT distance was 275 m, with a nadir oxygen saturation of 89% on room air and a heart rate recovery of 10 beats/min.

## Interpretation of Diagnostic Testing

Interstitial lung disease (ILD)-associated pulmonary hypertension (PH) is observed in a heterogenous group of fibrotic pulmonary parenchymal disorders, including idiopathic pulmonary fibrosis (IPF), combined pulmonary fibrosis with emphysema (CPFE), and connective tissue-associated ILD, among others.^1^^,^^2^ The prevalence of PH varies widely based on the underlying ILD cause and severity, as well as timing and method of screening. Still, severe PH is believed to be uncommon, occurring in 5%-10% of patients with fibrotic ILD.^2^, ^3^, ^4^ CPFE may be an exception to this rule, because PH may afflict up to one-half of all patients with CPFE, with severe PH being more common than in other fibrotic ILDs.^5^

The development of ILD-PH is associated with significantly increased morbidity and mortality relative to either ILD or PH alone.^2^, ^3^, ^4^ Patients with ILD-PH fare considerably worse than similar patients with pulmonary arterial hypertension (PAH) and no ILD, some estimates placing their mortality at over 3-fold greater compared with PAH.^3^ Similarly, mortality in patients with ILD-PH is much higher than in those with ILD alone, regardless of the cause of the underlying ILD.^2^^,^^4^ Together, this outsized clinical burden makes the development of ILD-PH a consequential event in patients with ILD, 1 that is unfortunately associated with an unfavorable long-term prognosis.

**Figure 2:**
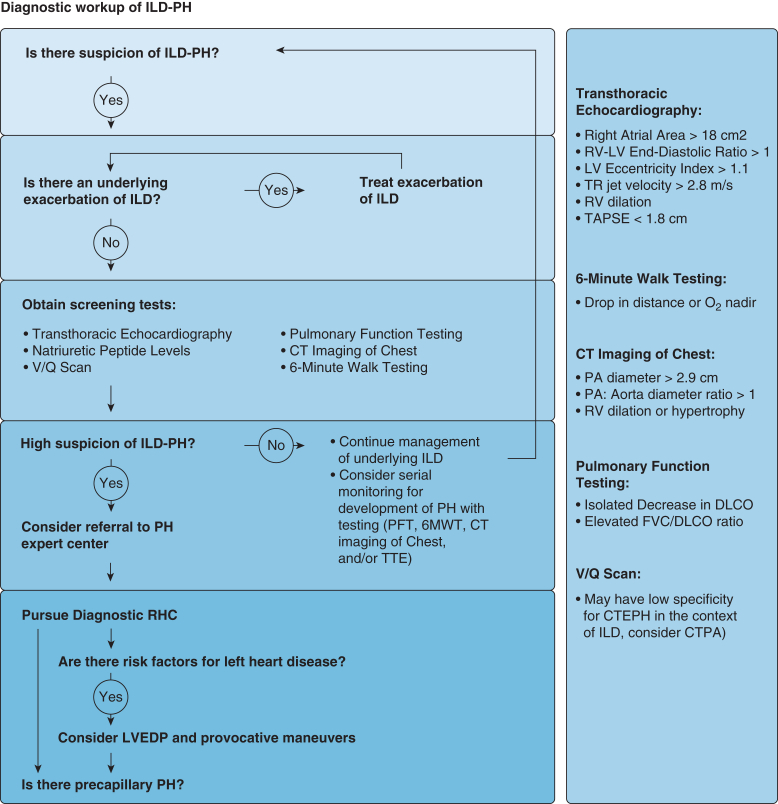
Diagnostic workup of ILD-PH. 6MWT = 6-minute walk test; CTEPH = chronic thromboembolic pulmonary hypertension; CTPA = CT pulmonary angiography; Dlco = diffusion capacity for carbon monoxide; ILD = interstitial lung disease; LV = left ventricle; LVEDP = left ventricular end-diastolic pressure; PA = pulmonary artery; PH = pulmonary hypertension; PFT = pulmonary function testing; RHC = right heart catheterization; RV = right ventricle; TAPSE = tricuspid annular plane systolic excursion; TR = tricuspid regurgitant; TTE = transthoracic echocardiography; $\dot{V}$/$\dot{Q}$ = ventilation-perfusion.

Because patients with ILD are at risk for developing various forms of PH, an accurate diagnosis requires ruling out other potential causes of PH (Fig 2). Because of common risk factors such as smoking exposure and age, patients with ILD often exhibit a high prevalence of associated cardiovascular comorbidities such as coronary artery disease, heart failure (systolic and diastolic), valvular heart disease, atrial arrhythmias, and sleep disordered breathing.^6^ These factors increase the risk for PH associated with left-sided heart disease. Patients with ILD are also at an increased risk of venous thromboembolism, predisposing them to chronic thromboembolic pulmonary hypertension.^7^ Furthermore, they may have comorbid connective tissue disease or HIV infection,^8^^,^^9^ which are independent risk factors for the development of PAH. Consequently, obtaining a comprehensive history is necessary to ensure accurate diagnosis and inform optimal management of ILD-PH. Referral to a PH center for support in obtaining and interpreting key diagnostic testing may be helpful.

Screening for PH using TTE can be challenging in patients with ILD because of poor acoustic windows and limited characterization of the tricuspid regurgitant jet envelope, which can make the RVSP unmeasurable in up to 50% of patients.^2^ Moreover, even when the regurgitant jet is well visualized, there is poor correlation between estimated RVSP and hemodynamically measured systolic pulmonary artery pressure, further limiting the utility of TTE screening for PH in the population of patients with ILD.^10^ The presence of abnormal right-sided heart morphology (RA dilation with area >18 cm^2^, RV dilation, RV/LV end-diastolic diameter ratio >1, LV eccentricity index >1.1) or RV dysfunction (subjectively assessed or objectively quantified by tricuspid annular plane systolic excursion or RV fractional area change) increases the likelihood of severe PH.^1^^,^^2^^,^^11^ CT imaging findings suggestive of PH (enlarged pulmonary artery > 2.9 cm, pulmonary artery to aorta diameter ratio >1, RV dilation, and hypertrophy) also may be helpful.^1^^,^^2^

Plasma BNP or N-terminal pro-BNP are established biomarkers that correlate with both pulmonary arterial pressures and mortality in PAH^1^^,^^12^ and may be helpful in screening for PH in ILD. Unfortunately, they are nonspecific and can be elevated in the context of underlying left-sided heart failure, renal failure, or ILD exacerbations.^13^^,^^14^

Results of PFTs and 6MWTs can be useful in raising the index of suspicion for underlying PH in patients with ILD.^15^, ^16^, ^17^, ^18^ Patterns such as an isolated decrease in Dlco with preserved FVC or an increase in the FVC/Dlco ratio across serial PFTs, or a drop in walk distance, oxygen saturation, or heart rate recovery on serial 6MWTs, should raise suspicion for ILD-PH and prompt additional testing.

PFTs are also valuable for quantifying the severity of restrictive disease in ILD but alone are insufficient to rule out significant parenchymal lung involvement. Patients with CPFE often demonstrate preserved or “pseudo-normalized” lung volumes (often with marked reductions in Dlco) as a result of concomitant restriction from ILD and hyperinflation from emphysema.^19^ High-resolution CT imaging is recommended to accurately assess and phenotype underlying parenchymal lung disease.^2^^,^^20^^,^^21^ It is important to recognize that acute ILD exacerbations can result in hypoxemia or hypercapnia, transiently raising pulmonary artery pressures because of pulmonary vasoconstriction. Hence, the evaluation to confirm the presence of ILD-PH should be conducted when patients are clinically stable.^22^^,^^23^

In recent years, there have been efforts to develop multimodal ILD-PH screening tools that incorporate combinations of these variables; however, thresholds and predictive performance can vary across different causes of ILD.^11^^,^^17^^,^^24^^,^^25^ For example, combining TTE with chest CT imaging can help evaluate the presence and severity of right-sided heart disease, while simultaneously characterizing the type and extent of ILD, enhancing screening and characterization of ILD-PH.^15^^,^^24^ Specifically in IPF, several screening tools incorporating 6MWT, PFT, and pulse oximetry have been developed and validated in large cohorts^26^^,^^27^ and may be helpful as a screening tool for ILD-PH. However, these tools remain unvalidated in other types of ILD; thus, their value in screening for ILD-PH outside of IPF is less clear.

In the case presented, several findings raise concern for underlying PH: progressive dyspnea on exertion, an impairment in Dlco on PFTs seemingly out of proportion to the degree of restriction, enlarged pulmonary artery on chest CT, dilation of right-sided heart chambers on TTE, and elevated plasma BNP levels. However, some testing may have been conducted during a post-viral exacerbation of her underlying ILD, and this patient’s TTE and 6MWT should be repeated once the ILD flare has resolved.

## Case, Part 2

Given the acute worsening of dyspnea, ground-glass opacities on a background of a probable usual interstitial pneumonia pattern on chest CT scanning, and the absence of concomitant heart failure exacerbation, the patient was diagnosed with an acute ILD exacerbation and treated with a course of oral glucocorticoids and empiric antibiotics.^28^ An immunologic assay for anti-nuclear antibodies was weakly positive at a titer of 1:80, but connective tissue disease serologies were negative. A hypersensitivity panel was also negative, and environmental exposure history was unrevealing. In the context of a repeat CT scan demonstrating resolution of ground-glass opacities but persistence of the underlying probable usual interstitial pneumonia pattern, she was given a clinical diagnosis of IPF and started on antifibrotic treatment. She noted a mild improvement in her exertional dyspnea, and she was now able to walk 325 m on room air with a nadir desaturation to 90%. Based on the TTE findings and persistent clinical suspicion for PH, she was referred to a PH center for further evaluation.

Her physical examination results remained unchanged. A repeat TTE showed persistent RA and RV dilation with subjective RV dysfunction. A repeat BNP remained elevated at 215 pg/mL. A ventilation-perfusion scan indicated multiple perfusion defects matched to the pulmonary parenchymal abnormalities seen on chest CT imaging. HIV and hepatitis C virus antibodies were negative.

## Interpretation of Further Diagnostic Testing

Despite improvement following treatment for an ILD exacerbation, there was still concern for underlying PH. Further evaluation with a right heart catheterization (RHC) was recommended by the PH center.

Because of older age and frequent cardiovascular comorbidities, approximately 20% of patients with ILD-PH have post-capillary or combined pre- and post-capillary PH.^29^ In patients with suspicion of concomitant left-sided heart disease (multiple cardiovascular comorbidities, advanced age, or TTE findings of left atrial enlargement, LV hypertrophy or diastolic dysfunction, or elevated E/e’ ratio), provocative testing (using either fluid or exercise challenge) may be necessary to “unmask” post-capillary PH.^30^^,^^31^ Although a fluid challenge may be easier to conduct in a practical setting, it is not without limitations, and provocative maneuvers have not been specifically validated in the ILD-PH population.

Although the ideal scenario is an accurate measurement of the pulmonary capillary wedge pressure (PCWP), this can be challenging in patients with ILD with considerable respiratory variation in intrathoracic pressures.^32^ Measuring wedged pulmonary artery catheter blood oxygen saturation to confirm appropriate catheter positioning and averaging the PCWP over several respiratory cycles (instead of using end-expiratory measurements) can help, but a discrepancy between measured and “true” effective wedge pressure may remain.^2^^,^^33^^,^^34^ Thus, in cases in which the accuracy of the PCWP is in question, or a reliable waveform is difficult to obtain, acquisition of a left-ventricular end-diastolic pressure may be informative in patients with ILD, particularly those with suspicion of post-capillary PH. Vasoreactivity testing is not recommended in the workup of ILD-PH.

## Case, Part 3

RHC testing revealed a right atrial pressure of 11 mm Hg, a pulmonary artery pressure of 67/23 mm Hg, a mean pulmonary artery pressure (mPAP) of 38 mm Hg. PCWP averaged across the respiratory cycle was 10 mm Hg (confirmed by wedge position oxyhemoglobin saturation), thermodilution cardiac output was 4.4 L/min, cardiac index 2.6 L/min/m^2^, and the calculated pulmonary vascular resistance (PVR) was 6.4 Wood units (WU).

## Management Approach

**Figure 3:**
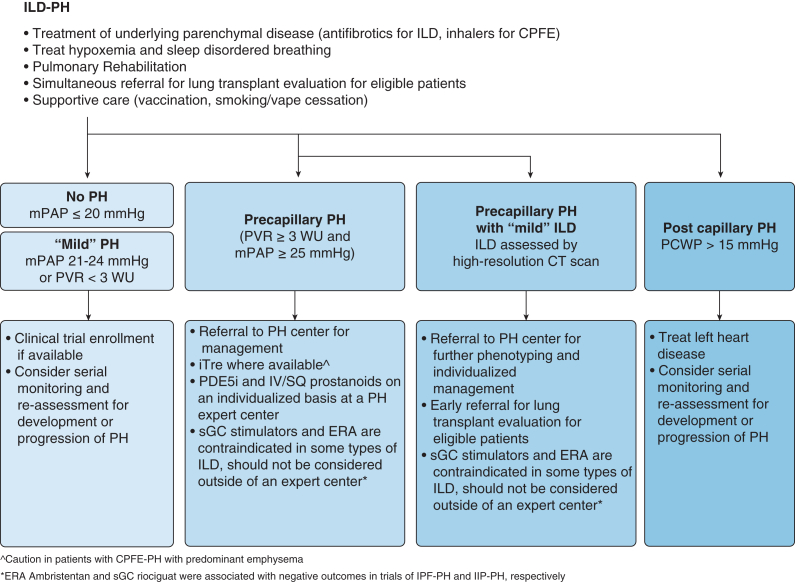
Management of ILD-PH. Abbreviations: CPFE = combined pulmonary fibrosis with emphysema; ERA = endothelin receptor antagonist; ILD = interstitial lung disease; iTRE = inhaled treprostinil; mPAP = mean pulmonary artery pressure; PCWP = pulmonary capillary wedge pressure; PDE-5i = phosphodiesterase-5 inhibitor; PH = pulmonary hypertension; PVR = pulmonary vascular resistance; sGC = soluble guanylate cyclase stimulator; SQ = subcutaneous; WU = Wood units.

Based on the most recent definition for precapillary PAH (mPAP > 20 mm Hg, PCWP ≤ 15 mm Hg, PVR > 2 WU) in the context of fibrotic ILD, a diagnosis of ILD-PH was made. In patients with ILD-PH, treatment of the underlying lung disease is of paramount importance and remains essential regardless of the presence or absence of PH (Fig 3). Because *all* patients with ILD-PH should be evaluated for both lung transplant suitability and clinical trial enrollment, referral to a PH center for management (if not already completed earlier) is strongly recommended.

The cornerstones of ILD therapy include smoking cessation, supplemental oxygen (if hypoxemic at rest, with activity, or with sleep), immunosuppressive and anti-fibrotic therapy for ILD (if appropriate), inhalers for concomitant obstructive lung disease, noninvasive ventilatory support for sleep disordered breathing and chronic hypercapnia (if indicated), pulmonary rehabilitation, respiratory illness vaccination, and lung transplant evaluation.^1^^,^^2^ In addition, any element of fluid overload should be treated with diuretics. These facets of care should be addressed as treatment of PH is considered in tandem.

When considering PH therapy in patients with ILD-PH, there is limited evidence from randomized controlled studies that included patients with hemodynamically diagnosed PH (mPAP ≥ 25 mm Hg and PVR > 3 WU) and no data to support treatment in those with “milder” PH (mPAP, 21-24 mm Hg; PVR, 2-3 WU).^1^^,^^2^

The Riociguat for Idiopathic Interstitial Pneumonia Associated Pulmonary Hypertension (RISE-IIP) trial randomized a cohort of participants with idiopathic interstitial pneumonia (IIP) with PH (most with IPF, notably excluding those with connective tissue disease-associated ILD) to the soluble guanylate cyclase stimulator riociguat or placebo, but was terminated early because of serious adverse events and higher mortality in the treatment arm.^35^ A subsequent post-hoc analysis suggested that mortality events may have been driven by participants with CPFE and with greater extent of emphysema relative to lung fibrosis on radiologic assessment.^36^ Likewise, the ARTEMIS trial, which randomized participants with IPF to the endothelin receptor antagonist ambrisentan or placebo, was terminated early because of an increased risk of respiratory hospitalizations and disease progression in the treatment arm.^37^ A sub-group analysis restricted to patients with IPF-PH showed similar signals for harm with ambrisentan. On the basis of these studies, soluble guanylate cyclase stimulators and endothelin receptor antagonists are not recommended for treatment of IIP-PH and IPF-PH, respectively, by current guidelines.^1^^,^^2^

The largest ILD-PH trial to date and the best evidence for treatment of ILD-PH comes from the Inhaled Treprostinil in Pulmonary Hypertension Due to Interstitial Lung Disease (INCREASE) trial.^38^ This trial randomized patients with idiopathic interstitial pneumonias such as IPF (approximately 50%), ILD associated with connective tissue diseases (approximately 25%), and CPFE (approximately 25%) 1:1 to inhaled treprostinil (iTRE) or placebo. Treatment with iTRE not only improved functional capacity measured by 6MWT distance but also improved BNP levels and reduced clinical worsening. Prespecified analyses indicated that the greatest benefit was observed in participants who achieved a dose of 9 or more breaths 4 times per day and had a baseline PVR ≥ 4 WU. Post-hoc analyses also suggested iTRE treatment stabilized pulmonary function (FVC) and diminished the risk of ILD exacerbations and clinical worsening vs placebo.^39^, ^40^, ^41^

When considering CPFE-PH, treatment should be approached on an individual basis. Notably, prespecified analyses of the INCREASE trial showed that those with CPFE-PH did not derive the same 6MWT distance benefit as those with other types of ILD.^38^ However, in a non-prespecified post-hoc analysis, patients with CPFE-PH did have fewer events of disease progression after a primary endpoint was met, suggesting the therapeutic benefit is maintained across all studied ILD subgroups.^38^^,^^40^ In contrast, the Inhaled Treprostinil in Pulmonary Hypertension Associated With COPD (PERFECT) trial, which randomized participants with COPD and PH to iTRE or placebo, was terminated early because of increased risk of serious adverse events and mortality in the treatment group, particularly those with a Dlco < 25% of predicted.^42^ Harmonizing this data from PERFECT and INCREASE, we recommend reserving iTRE for patients with CPFE with a predominantly fibrotic phenotype and limited emphysema, and caution against iTRE in those with heavy emphysema burden.

Aside from iTRE, parenteral prostacyclin therapy has been described as a treatment for ILD-PH with significant RV failure in case reports and small case series.^43^^,^^44^ If parenteral prostacyclin therapy is considered, it should be done on an individual basis in experienced PH centers after an appropriate and comprehensive discussion of risks and benefits with the patient. Given its importance as a cornerstone of ILD-PH management, lung transplant should be considered in *all* patients with ILD-PH, including those whose condition fails to respond to standard therapies and who are being considered for nonstandard therapies such as parenteral prostacyclins.

Globally the most readily accessible and affordable PH medications are phosphodiesterase-5 inhibitors (PDE-5is; sildenafil and tadalafil). Retrospective studies, registry analyses, and meta-analyses suggest that PDE-5i therapy may improve hemodynamics and survival in patients with ILD-PH with significant PH (PVR > 5 WU).^45^, ^46^, ^47^ Because there are no randomized controlled trials of PDE-5i therapy in ILD-PH, current guidelines support PDE-5i therapy in patients with ILD-PH when iTRE is unavailable, on an individualized basis, after careful discussion with patients, and while diligently monitoring for side effects.^1^^,^^2^

When considering ILD-PH, recognize that some patients have relatively mild ILD but significant PH. Real world data from the Comparative, Prospective Registry of Newly Initiated Therapies for Pulmonary Hypertrension (COMPERA) and Assessing the Spectrum of Pulmonary Hypertension Identified at a Referral Center (ASPIRE) registries describe this cohort of patients, presumptively carrying a diagnosis of idiopathic PAH (IPAH) but with evidence of interstitial lung abnormalities on CT imaging, whose outcomes mirror those of patients with ILD-PH.^21^^,^^48^^,^^49^ These patients are typically older and male, with a lower Dlco and more prevalent smoking compared with those with IPAH. Because there is no standardized definition of what constitutes “mild” ILD (thresholds of < 20% fibrosis on chest CT imaging or FVC > 70% have been suggested), and how it may vary by ILD cause, this entity is challenging to both identify and manage. Furthermore, recent registry studies suggest these patients have poor treatment response compared with patients with IPAH.^21^ In our opinion, these patients should be referred to a PH center for management. Given their high morbidity and mortality, and in the absence of additional data regarding prognosis and management, our treatment approach in patients with “mild” ILD and significant PH mirrors existing PAH treatment algorithms, with slight modifications to account for their underlying ILD: avoidance of endothelin receptor antagonists and soluble guanylate cyclase stimulator therapies, concomitant referral for lung transplantation consideration in appropriate patients, and referral to palliative care in those who are not transplant candidates.^2^

## Case, Part 4

Applying this knowledge to this patient, we note ILD-PH with a predominantly fibrotic phenotype in an appropriate candidate for iTRE. In the case of further deterioration, we could consider the addition of PDE-5i therapy after discussion of risks and benefits with the patient. Given the significant mortality of patients with ILD-PH, and in light of this patient’s age and absence of significant comorbidities, she should also be referred for pulmonary rehabilitation and to a lung transplant center for further evaluation.

It is important to recognize the case we presented here was purposefully straightforward to emphasize the approach to a patient suspected of ILD-PH. In typical clinical practice, where patient presentations are often more nuanced and complicated, with a subtler suggestion of PH, early consultation and referral to a PH center can be invaluable in supporting accurate screening, diagnosis, and management of these complex patients.

## Summary

ILD-PH is often encountered in clinical practice but is uniquely challenging to diagnose and manage successfully. Morbidity and mortality are high, the appropriate interpretation of diagnostic testing is complex, and PH centers are essential when navigating treatment decisions. In this edition of “How I Do It,” we provide a valuable framework for approaching the patient with suspected ILD-PH, while also identifying current knowledge gaps that need to be addressed to optimize the care of these challenging patients.

## Key Points

• Rigorous phenotyping, including physiologic testing (such as PFTs) and chest imaging (such as high-resolution CT), is essential to quantify the burden of parenchymal lung disease and ensure accurate diagnosis and management.
• Evaluation must take place when the patient is clinically stable, because ILD exacerbations can transiently increase pulmonary arterial pressures and confound the diagnosis.
• In patients with a high pretest probability of post-capillary or combined pre- and post-capillary PH, consider provocative maneuvers (fluid or exercise challenge) during RHC to unmask post-capillary PH. Consider left heart catheterization with left-ventricular end-diastolic pressure if PCWP accuracy is in question or difficult to obtain. Referral to PH centers is suggested for support with diagnostic testing and interpretation.
• Management of ILD-PH is multifaceted, including treatment of the underlying lung disease, correction of hypoxemia, addressing comorbidities and deconditioning, consideration for lung transplantation, and determining treatment of PH. Management at PH centers is emphasized.
• iTRE therapy has shown benefit in fibrotic ILD-PH, but should be avoided in CPFE-PH with a significant emphysema phenotype.
• In carefully selected ILD-PH cases, PDE-5i and parenteral prostacyclin therapy may be helpful, but should be considered on an individualized basis at an expert center.

## Funding/Support

This study is supported by an 10.13039/100000002National Institutes of Health grant (1K23HL16497) (A. J.).

## Financial/Nonfinancial Disclosures

The authors have reported to *CHEST* the following: A. J. reports serving on the consultant or advisory board of Janssen and Merck, has received research funding from United Therapeutics. N. S. has served on the speakers bureau for Bayer and has received research grant support from United Therapeutics and Insmed. J. M. E. has received research grant support from Janssen, United Therapeutics, Liquidia, Gossamer Bio, Bayer, Merck, Altavant, Aerovate, Pulmovant, and serves on the consultant or advisory board of United Therapeutics, Altavant, Aerovate, Pulmovant, Bayer, Gossamer Bio, Liquidia, Merck, and Janssen. B. A. has served on the consultant or advisory board and speakers bureau for Janssen. R. B. has served on the consultant or advisory board for Merck, Janssen, United Therapeutics. A. R. T. has served on the consultant or advisory board for Janssen and Merck. R. A. E. has served on the consultant or advisory board and speakers bureau for United Therapeutics, Janssen, and Merck, and has served on the consultant or advisory board for Gossamer Bio and Liquidia. J. V. has served as a medical expert witness, on the DSMB for Syneos, and is supported by an NIH grant (U34HL147347: PE-TRACT trial). S. S. has served on the consultant and advisory board for Merck, Janssen, and United Therapeutics, has received research support from United Therapeutics, Keros, Liquidia, and Pulmovant, and directs the pulmonary hypertension program at the Houston Methodist Hospital supported by the Charles and Diane PH Fund. O. A. S. serves on the steering committee for Gossamer, Insmed, and AllRock, has served on the consultant or advisory board of Merck and United Therapeutics, and has served on the speakers bureau for United Therapeutics. None declared (A. B., M. S., F. J. S., D. V.).
